# The Treatment of Neurasthenia

**Published:** 1904-09-24

**Authors:** 


### THE TREATMENT OF NEURASTHENIA.

Dr. Chas. W. Buckley[1] divides cases of neurasthenia into (1) those due to shock, as *e.g.* traumatism, (2) those due to nerve exhaustion, and (3) those due to nerve-starvation, auto toxæmia, etc. In many cases causes 2 and 3 are both in operation. The general attitude of the physician towards the neurasthenic patient should be "sympathetic without sympathising." Indifference towards his story of subjective sensations will only destroy his confidence, and exhortations to "make an effort" are worse than useless. In the forefront of treatment stands the removal of the patient from the strain or worry responsible for his breakdown; this in most cases means withdrawal from home life and his usual surroundings. In a few mild cases, resulting from overwork, a sea voyage works wonders, but in the majority of cases it does harm. Much the same may be said of travel. The need usually is for bodily as well as mental rest, and exceptionally when milder measures fail the discipline of the Weir-Mitchell treatment may be needed; if this is adopted it should be applied thoroughly, and not in any of the so-called "modified" forms. Residence at a moderately high level and some little distance inland rather than on the coast is the most suitable. If the patient cultivates fishing, golf, etc., he may do well in some quiet spot, but otherwise a health resort with its scheme of entertainment will be advisable. Another element in treatment is the correction of organic or functional defects in any part of the body; for example, astigmatism, naso-pharyngeal abnormalities, sexual disturbances, menstrual disorders, gastric and intestinal irregularities, septic conditions of the teeth, gums, etc. In some instances, and when milder measures prove ineffective, operation for such a condition as movable kidney, hæmorrhoids, etc., may be necessary. A third aspect of treatment is suggested by vaso-motor disturbances, which may find expression in visceral engorgement, as, for example, hepatic disorders. Here hydro-therapy has a large function. Daily cold baths, or, with less vigorous patients, hot baths, followed by cold affusion to the neck and spine are of great tonic value. A towel wrung out of strong salt solution allowed to dry and then used for friction of the whole body is also beneficial. As sedatives wet-sheet packs are very efficacious. Massage is another method of overcoming vaso motor disturbances, and resisted movements improve the function both of the heart and the blood-vessels. Galvanism, too, is often useful. In the form of the electric bath it stimulates nutrition, but the sinusoidal or alternating dynamo-current is more generally useful; it is more stimulating, pleasanter, and less exhausting to the patient. In various abdominal conditions as splanchnic stasis, constipation, etc., galvanism may be applied by means of a douche-electrode inserted into the rectum which is distended with saline solution. Faradic massage exerts a valuable stimulant action on the nerves of the skin and on the nutrition of the deeper structures. The fourth object in treatment is to secure the nutrition of the nervous tissues. The food should be readily digestible and should contain abundance of phosphorus. In the early stages of cases marked by intestinal indigestion and auto-intoxication the Salisbury diet—though rarely in its most rigid form—may be adopted for a time. Organic combinations of phosphorus as the glycerophosphates and lecithin are useful, though where cheese, eggs, etc., can be well digested abundance of the element can be supplied in the form of food substances. In the treatment of special symptoms headache and sleeplessness call for consideration, and may need drug treatment if the physical methods applied for the general condition fail. Strychnine as usually given is little more than a whip to a tired horse, but in smaller doses it improves nerve nutrition. Arsenic is of value in gastric cases; bromides are best avoided if possible; valerian may produce temporary benefit; and supra-renal gland may improve symptoms due to vaso motor paresis. But in all cases drugs occupy a position entirely subordinate to other methods of treatment.

[1] Practitioner, August, 1904.

